# Burden and distribution of venous thromboembolism across cancer types and stages: a meta-analysis of observational studies

**DOI:** 10.3389/fonc.2025.1619554

**Published:** 2025-09-30

**Authors:** Vikram Singh, Heena Tabassum, Simran Kohli, Peteneinuo Rulu, Sumit Aggarwal

**Affiliations:** ^1^ Division of Descriptive Research, Indian Council of Medical Research, New Delhi, India; ^2^ Division of Non-communicable Diseases, Indian Council of Medical Research, New Delhi, India

**Keywords:** VTE, cancer, thrombosis, cancer types, cancer stages, neoplasm

## Abstract

**Introduction:**

Venous thromboembolism (VTE) is a serious life-threatening complication among patients with cancer. This systematic review and meta-analysis aimed to quantify the proportion of VTE across different cancer types, stages, and study settings.

**Methods:**

A comprehensive literature review was conducted to identify observational studies reporting VTE events in cancer patients. Studies were categorized into population-based and hospital-based settings to compare VTE prevalence. Cancer types were classified by anatomical origin, and cancer stages (Stage I–IV). Meta-analytic techniques were applied using R software, and pooled VTE proportions with 95% confidence intervals (CIs) were calculated. Heterogeneity was assessed using the I² statistic, and forest plots were generated for visualization. Sensitivity analyses were conducted to evaluate the robustness of findings.

**Results and discussion:**

The analysis included 14 studies, out of which hospital-based studies reported a significantly higher VTE proportion (4.1%) compared to population-based studies (2.9%) (*p* <.001, χ² = 1452.219). Among cancer types, gastrointestinal malignancies had the highest pooled VTE proportions, followed by hematologic and urogenital cancers. In hospital-based cohorts, lung and breast cancers showed particularly high VTE burdens. VTE risk increased with advancing cancer stage, with the highest proportions observed in Stage III and IV cancers. This meta-analysis demonstrates substantial variation in VTE risk based on study setting, cancer type, and stage. The findings underscore the need for nuanced, type- and stage-specific VTE risk assessment models to guide effective prophylaxis and clinical decision-making in oncology practice.

**Systematic review registration:**

https://www.crd.york.ac.uk/PROSPERO/view/CRD42024616005, identifier CRD42024616005.

## Introduction

Venous thromboembolism (VTE) is a condition characterized by the formation of blood clots within the venous system. It comprises two major clinical manifestations: deep vein thrombosis (DVT) and pulmonary embolism (PE) (1). Among patients with cancer, VTE is a common and critical complication, often associated with significant morbidity and mortality. Cancer patients face a significantly elevated risk of VTE compared to the general population, with estimates indicating a nine-fold increase in risk (2). This heightened risk is compounded by higher rates of bleeding and recurrence during anticoagulant therapy in cancer patients, underscoring the complex clinical management of this population. Since its first identification in 1823 (3), cancer-associated VTE has remained a critical challenge in oncology, linked to treatment interruptions, reduced quality of life, increased healthcare costs, and poorer overall survival (4, 5).

Multiple large-scale studies and reviews have highlighted the disproportionate VTE burden in specific malignancies. Khorana et al. (2007) (6) and Ay et al. (2010) identified pancreatic, gastric, lung, and brain cancers among the most thrombogenic tumors (7). Mechanistic studies have attributed this to tumor expression of procoagulant proteins such as tissue factor (TF), cancer cell-derived microparticles release, and coagulation cascade activation. Furthermore, studies by Lee et al. (2003) (8) and Louzada et al. (2011) demonstrated that advanced-stage disease is associated with a higher risk of initial and recurrent thrombotic events. Despite these insights, few reviews have provided pooled, comparative estimates stratified by cancer type, stage, and healthcare setting. This hinders the development of cancer-specific VTE risk stratification tools that are applicable in real-world clinical practice (9).

The global burden of VTE among cancer patients is significant, with substantial heterogeneity in its presentation across populations and healthcare systems (10). Advances in cancer therapies and diagnostic imaging have contributed to improved survival rates in cancer patients but may have also influenced the incidence and burden of cancer-associated VTE (11). Novel therapies, including immunotherapies and targeted treatments, introduce additional thrombotic risks, necessitating an updated understanding of VTE in the evolving oncology landscape (12). Biomarkers such as elevated D-dimer and Factor VIII levels have emerged as valuable predictive tools for assessing VTE risk, providing insights into patient stratification and prophylactic decision-making (13).

Despite advancements in treatment, cancer-associated VTE continues to pose significant challenges. Research has also highlighted that VTE remains a serious complication for cancer patients, even with substantial improvements in oncology care (14). Small-scale studies have further identified VTE as one of the leading causes of death in cancer patients (15). The consequences of VTE extend beyond physical health, impacting patients’ psychological well-being and imposing a substantial economic burden on healthcare systems (16).

Given the complexity of cancer-associated VTE, current clinical guidelines emphasize the importance of early identification and thromboprophylaxis in cancer patients at elevated risk (17). However, the risk of VTE is not uniformly distributed across all cancer patients; it is known to vary significantly depending on cancer type, biological behavior, and the stage of disease progression (13). For instance, certain malignancies might be consistently associated with higher thrombotic risk, while advanced-stage cancers are more likely to trigger systemic prothrombotic responses due to increased tumor burden, vascular invasion, and treatment intensity. Despite these known patterns, there remains a lack of consolidated, comparative evidence that stratifies VTE burden across both cancer types and clinical stages using pooled observational data. In light of this variability, the present systematic review and meta-analysis seeks to fill this gap by quantifying the global proportion of VTE among cancer patients, stratified by study setting, cancer type, and stage.

### Research question

The researchers aimed to conduct a systematic review and meta-analysis related to the development of VTE as a medical condition among patients diagnosed with cancer. The primary research question of this review was:

What is the proportion of venous thromboembolism (VTE) among patients with different types and stages of cancer?

## Methods

### Data sources and searches

This review was conducted and reported in accordance with the Preferred Reporting Items for Systematic Reviews and Meta-Analyses (PRISMA) guidelines. During the scoping phase, previously published systematic reviews and meta-analyses were consulted to identify evidence gaps, refine cancer-type classifications, and inform the development of the search strategy. However, these secondary sources were not included in the formal selection process, in line with the review’s focus on primary data.

A comprehensive search was conducted on PubMed between 2nd and 12th December 2024 to identify studies reporting quantitative estimates of VTE incidence in cancer patients across different stages. The search strategy was developed through an iterative process, incorporating both free-text terms and Medical Subject Headings (MeSH), and used Boolean operators (AND/OR) to enhance precision and coverage. The final search string was: ((cancer OR tumor OR malignancy OR cancer patients) AND (Venous Thromboembolism OR venous thromboembolism OR VTE OR thrombosis OR thromboembolism) AND (risk OR risk factors OR predictors OR treatment-related risk OR clinical risk factors)).

The search was limited to English-language, full-text studies involving human participants, thereby excluding animal-based research. The date range was restricted to studies published between 2000 and December 2024, capturing developments over the last 25 years. This period was selected for both clinical and methodological reasons. Notably, the 6th edition of the TNM Classification of Malignant Tumors, introduced in 2002 (18), brought standardization to cancer staging, essential for stratifying VTE risk by stage. The early 2000s also marked advances in cancer therapies, increasing awareness of cancer-associated VTE, and the implementation of standardized thromboprophylaxis guidelines. Restricting the review to this timeframe ensured inclusion of studies aligned with modern oncology practices and diagnostic frameworks, thereby enhancing comparability and clinical relevance.

The search strategy primarily targeted studies reporting cancer-associated VTE incidence or proportions. Although the term “risk factors” was included to improve sensitivity and capture observational data, this may have biased retrieval toward analytical studies. To maintain alignment with the review objective, we applied strict eligibility criteria during screening, including only studies reporting VTE incidence or proportion stratified by cancer type or stage.

### Study selection

Three reviewers SK, VS, and, SA reviewed titles, abstracts, and full-text articles, with any discrepancies about study inclusion resolved by discussion among all three authors. Rayyan QCRI (19) was used to identify potentially eligible articles and remove duplicates, if any, to minimize the occurrence of selection bias. This review included studies that specifically investigated cancer-associated VTE in patients of any age or sex. Only observational studies were included, as they are particularly well-suited to capturing real-world incidence patterns of VTE across diverse clinical settings and patient populations, including variations by cancer type, stage, and geographic location (20). To ensure the broadest possible representation, studies published in English were included without geographic restrictions. Studies were excluded if they were case reports, conference abstracts, opinion pieces, editorials, or review articles that did not include original or primary data. Additionally, research with insufficient or incomplete reporting of VTE incidence data was also excluded.

**Figure 1:**
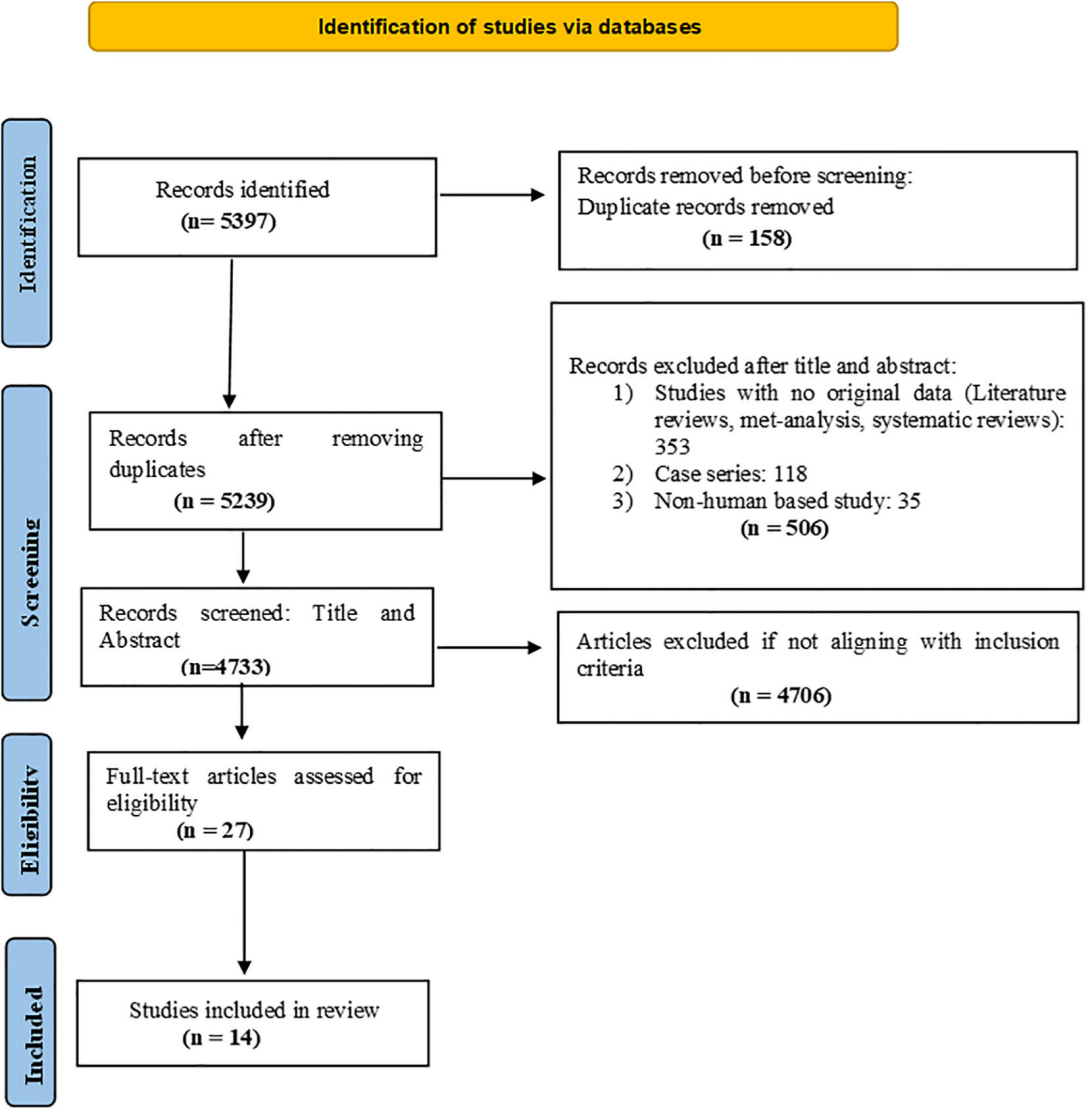
Prisma flow chart.

The review protocol was registered in the PROSPERO International Prospective Register of Systematic Reviews (CRD42024616005) and the search results have been depicted in the form of a flow diagram (**Figure 1**) as recommended by the PRISMA – Preferred Reporting Items for Systematic Reviews and Meta-analysis guidelines.

### Data extraction

Initial data extraction was performed by one reviewer, followed by a blinded, independent review by at least one additional team member. Any discrepancies were resolved through consensus discussions among the full review team. While simultaneous dual extraction was not performed, this sequential cross-checking approach ensured rigorous quality control and minimized the risk of bias or errors. The following data were extracted author, title, year of publication, country, objective, sampling, study design, participant characteristics, and quantitative results (type and stage of cancer, VTE rate). **Table 1** depicts the basic characteristics of all the selected studies.

**Table 1:** Characteristics of included studies.

First author (reference)	Country	Date of publishing	Type of study	Number of study participants	Gender (M/F)	Age (mean/median, range or grouped)	Number of VTE patients	Cancer type	Cancer stage
Ikeda et al. (21)	Japan	2022	Hospital Based	2477	1407/1070	Mean age: 68.1	158	Colorectal cancer (2477)	II, III, IV
Dimakakos et al. (22)	Greece	2021	Hospital Based	217	176/41	Mean age: 67.8	9	Small cell lung cancer (217)	II and IV
Khorana et al. (13)	USA	2007	Hospital Based	1,015,598	521991/493583	Grouped: <65: 573,645, >65: 441,953	45,872	Breast (70,917), Stomach (15,934), Colon (42,258), Pancreas (26,118), Bladder (23,584), Kidney (29,651), Prostate (92,584) Testes (3,687), Brain (35,297), Sarcoma (21,989), Non-Hodgkin lymphoma (56,964),, Myeloma (21,804), Leukemia (46,977), lung (107,587), Ovary (23,839)	Not Available
Matsuoa et al.	USA	2020	Hospital Based	798	Only female	Mean age: 48.9 years	98	Cervical cancer (798)	I, III, IV
Al Diab et al. (23)	Saudi Arabia	2010	Hospital Based	306	111/195	Grouped: 13-25: 26, 25-45: 123, 45-60: 95, 60-80: 45, >80: 17	43	Breast cancer (165), Colon cancer (73), lung cancer (45), and sarcoma (23)	I–IV
Vormittag et al. (24)	Austria	2009	Hospital Based	840	462/378	Mean age: 62 years	62	Brain (103), Breast (136), Lung (120), Upper gastrointestinal (36), Colorectal (111), Pancreas (47), Kidney (24), Prostate (105), Multiple myeloma (17)	II and IV
Saadeh et al. (25)	Ireland	2013	Hospital Based	344	Only female	Median age: 57	33	Ovarian Cancer (344)	I–IV
Mandala et al.	Italy	2009	Hospital Based	381	119/262	Median age: 62	30	Breast (182), pancreas (10) and colon (122)	I–IV
Tagalakis et al. (26)	Canada	2007	Hospital Based	493	297/196	Mean age: 64.5 years	67	Non-small cell lung cancer (493)	I and II
Bloma et al.	Netherland	2005	Hospital Based	202	115/87	Mean age: 64 years	19	Pancreatic Cancer (202)	I-IV
Grilz et al.	Austria	2024	Population Based	8306244	4255119/4051125	Median age: 41	15803	Gastrointestinal (35,490), Mesothelium/soft tissue (3,897), Breast (23,164), Male genital (21,232)	Not Available
Balabanova et al. (27)	Sweden	2022	Population Based	92 105	Only males	Median age: 69	2955	Prostate cancer (92,105)	I-IV
Marks et al. (28)	USA	2014	Population Based	1138390	60433/534057	Grouped: 65-69: 192272, 70-74: 296027, 75-79: 287109, 80-84: 205451, ≥ 85: 157531	3459.75	Lung cancer (179,880), stomach cancer (22,860), colon cancer (107,265), gallbladder cancer (3777), pancreatic cancer (33175), soft tissue cancers including heart, melanoma, ovary cancer (16,112), and kidney/renal pelvis cancers (24,611), non-Hodgkin lymphoma (55,195), myeloma (15,318), and acute myeloid leukemia (AML, 8,489), mesothelium (4728)	I-IV
Van Hemelrijck et al. (29)	Sweden	2010	Population Based	76600	Only males	Grouped: <65: 2941, 65-74: 9255, >75: 18446	1881	Prostate Cancer (76600)	I-IV

### Quality assessment

The Joanna Briggs Institute (JBI) tools were chosen for assessing the quality of the included studies (JBI Critical Appraisal Tools). JBI offers a range of tools tailored to different study designs, ensuring appropriate evaluation criteria. The JBI tools provide a comprehensive set of criteria for evaluating the methodological quality of studies. These criteria typically cover aspects such as study design, participant selection, intervention description, outcome measurement, statistical analysis, and risk of bias assessment. The JBI tools typically provide response options such as “Yes,” “No,” “Not Applicable (N/A),” and “Unclear” for each assessment criterion. Reviewers selected the most appropriate response based on their evaluation of whether the study adequately addressed the criterion.

### Data analysis

The analytical approach was structured across three levels to comprehensively evaluate the proportion of VTE among cancer patients. The first level of analysis aimed to compare population-based and hospital-based studies to assess setting-specific differences in venous thromboembolism (VTE) prevalence. A population-based study draws its sample from an entire defined population or community, such as residents of a geographic region or members of a national registry (30). The goal is to obtain a representative snapshot of the population, allowing for prevalence or risk estimates that are broadly generalizable. In this meta-analysis, population-based studies refer to those that utilized large national health registries or administrative databases to evaluate VTE risk among cancer patients. Such studies offer broad, generalizable estimates across both inpatient and outpatient settings (27–29, 31).

In contrast, a hospital- based study selects its sample from patients who visit a specific healthcare facility or group of facilities. While it may provide detailed clinical insights, its findings are less generalizable due to a narrower, treatment-seeking population (30). In the present review, studies included in this category were conducted within clinical settings, such as oncology departments or tertiary care centers, and focused on patients undergoing active cancer treatment. These studies relied on institutional records to document VTE events occurring during hospitalization or therapy, thereby capturing more granular data on treatment-related risks (6, 16, 24). This comparison was designed to capture potential variations attributable to disease severity, clinical monitoring, and treatment exposure, which are typically more pronounced in hospital settings than in population-based cohorts.

At the second level, analyses were performed based on cancer type, and for this, cancers were systematically classified into broad categories according to their anatomical origin. The classification includes gastrointestinal cancers, which have malignancies of the digestive system, such as colorectal, stomach, pancreatic, and gall bladder cancers, and Urogenital Cancers encompassing tumors arising from the genitourinary system, including prostate, kidney, testicular, and ovarian cancer. Also, Hematologic malignancies involve blood-related cancers, such as non-hodgkin lymphoma, myeloma, and leukemia/acute myeloid leukemia. Other tumors were retained as independent classifications due to their varied pathophysiology, these include sarcoma, breast, brain, mesothelium and, and lung cancers (see **Supplementary Tables 6**, **7**).

The third level of analysis was based on cancer stage. Since the included studies employed heterogeneous staging terminologies. While the standard TNM classification system (Tumor size, Node involvement, Metastasis) serves as the gold standard for cancer staging, several studies in this meta-analysis provided only partial TNM data. Therefore, a harmonization process was undertaken to map cancer stages into four standard categories: Stage I (Localized), Stage II (Early Locally Advanced), Stage III (Late Locally Advanced), and Stage IV (Metastatic). The details of this harmonization process are outlined in **Supplementary Table 8** (**Appendix**).

Statistical analyses were conducted using R software version 36.0 (R Foundation for Statistical Computing) to evaluate the proportion of VTE in cancer patients. Pooled VTE proportions were calculated along with 95% confidence intervals (CIs) to quantify the estimated risk across different cancer types. Heterogeneity among studies was assessed using the I squared (I²) statistic, with thresholds of 25%, 50%, and 75% indicating low, moderate, and high heterogeneity, respectively. To visually represent the meta-analytic findings, forest plots were generated to illustrate individual study estimates, pooled prevalence rates, and overall heterogeneity. These visualizations were produced using the meta packages in R, facilitating a comprehensive depiction of effect estimates across cancer types. Furthermore, sensitivity analyses were carried out by sequentially excluding individual cancer subtypes to examine their influence on the overall pooled VTE estimates and heterogeneity statistics. This approach enabled the assessment of the robustness of the findings and the identification of cancer types that contribute disproportionately to between-study variability. Statistical significance was set at *p* < 0.05, and all analyses were conducted in accordance with established meta-analytic guidelines to ensure methodological transparency and reliability.

## Results

### Selection of studies

A rigorous selection process was followed in accordance with PRISMA guidelines. Initially, 5,397 records were identified through database searches. After removing 158 duplicates, 5,239 unique records were screened for relevance. During the title and abstract screening phase, 506 records were excluded for the following reasons: lack of original data (e.g., literature reviews, meta-analyses, systematic reviews), case series, animal-based studies, or surveys. Studies that did not report VTE occurrence stratified by cancer type or stage, or those in which VTE was not the primary outcome in cancer patients, were also excluded at this stage. This resulted in 4,733 records being considered for further evaluation. Subsequently, 4,706 records were excluded for not meeting the inclusion criteria. The remaining 27 full-text articles were assessed for eligibility. Of these, 13 were excluded after full-text review, resulting in 14 studies that fulfilled all inclusion criteria and were included in the final review. We included observational studies that reported the incidence or proportion of VTE in cancer patients, provided the data were stratified by cancer type or stage. Cross-sectional studies were excluded only if they did not specifically investigate cancer-associated VTE or failed to present stratified data relevant to our research objective. To minimize ambiguity, studies were not excluded solely based on their use of the term “prevalence” to describe VTE outcomes.

### Overview of included studies

**Figure 2:**
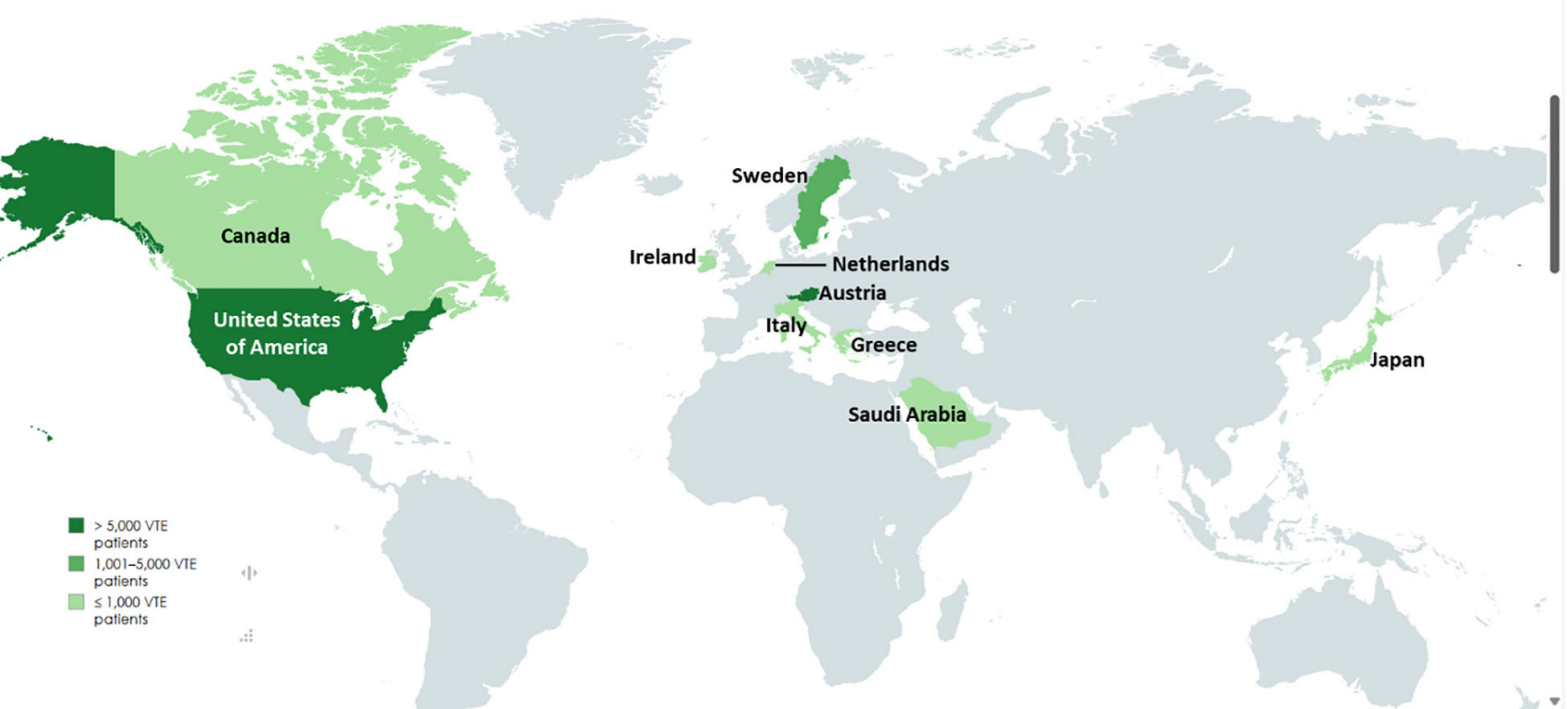
Geographical distribution of the studies included; the grading shows the population size included in the studies from the respective country. (Created in mapchart.net).

The 14 studies included in this systematic review span multiple continents and employ various study designs to examine venous thromboembolism (VTE) in cancer patients. A summary of the included studies is presented in **Table 1**. These studies were conducted in a diverse range of countries, including Japan (21), Austria (31), Greece (22), the United States (6, 24), Saudi Arabia (23), Sweden (27, 29), Ireland (25), Canada (26), Italy (32), and the Netherlands (33), thereby contributing to a globally representative dataset. The geographical distribution of these studies is illustrated in **Figure 2**, where the grading reflects the number of studies reported per country, based on the number of VTE cases.

The studies employed two principal research designs: hospital-based and population-based (**Table 1**). Of the 14 studies, 10 followed a hospital-based design (6, 21–26, 32, 33), focusing on patient-level data within clinical or tertiary care settings. The remaining four were population-based studies that utilized national or regional healthcare registries (27–29, 31).

### Risk of bias assessment

The JBI critical appraisal tool was employed to evaluate the risk of bias in the included studies. The assessment was conducted based on a set of methodological criteria outlined in the tool. Based on the JBI cohort checklist, the risk of bias assessment across 13 included cohort studies reveals a generally favorable methodological quality, especially in domains concerning population selection and exposure assessment. Most studies received a “Yes” rating in the early domains (D1–D5), suggesting that participants were appropriately selected, exposures were reliably measured, and confounders were identified. However, issues arise in later domains related to follow-up and outcome management. Several studies, such as Grilz et al. (2024) and Mandala et al. (2009), demonstrated high risk of bias, with “No” ratings particularly in domains assessing follow-up completeness and statistical adjustments (D9 and D10). A few studies, including Diab et al. (2010) and Balabanova et al. (2022), had multiple “Unclear” ratings in the follow-up and outcome domains (D6–D10), indicating potential concerns about reporting transparency or study design limitations. In contrast, Bloma et al. (2005) and Hemelrijck et al. (2010) showed consistently low risk across almost all domains. Overall, while the majority of the studies were methodologically robust in their design and reporting, select studies with unclear or high-risk ratings in key areas should be interpreted with caution during evidence synthesis.

**Figure 3:**
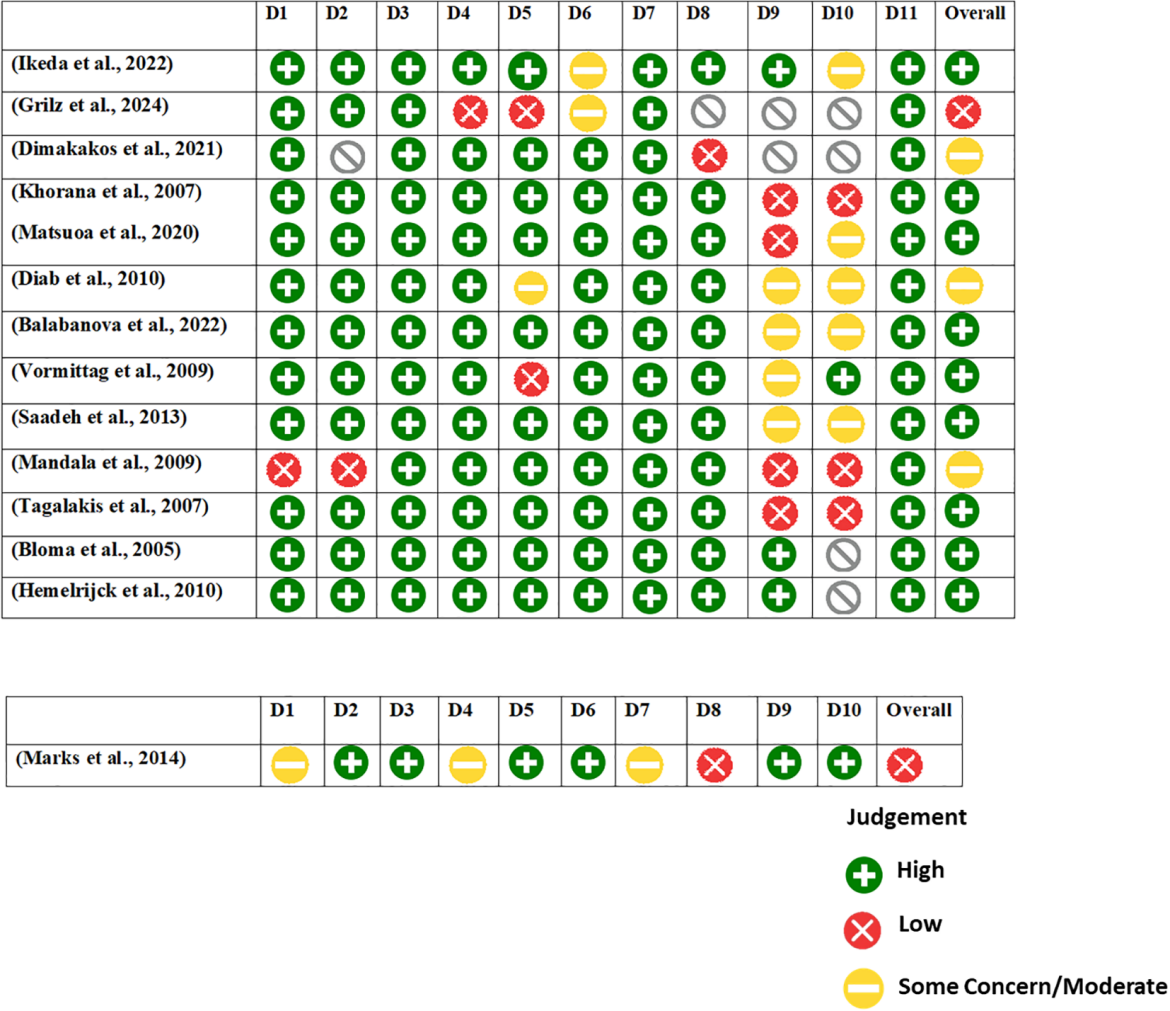
Risk of bias according to the items of the JBI checklist.

The risk of bias assessment for the case-control study by Marks et al. (2014), based on the JBI checklist, reveals moderate risk. The study performed well in consistent exposure measurement (D3, D5), identification of confounding factors (D6), and use of appropriate statistical analysis (D10). However, concerns arise due to unclear group comparability (D1), exposure assessment (D4), and strategies for managing confounding (D7). Notably, outcome assessment (D8) was inadequate (“No”). These limitations suggest potential bias, warranting cautious interpretation of the findings (**Figure 3**).

## Meta-analysis

### VTE proportion in population and hospital-based studies

Among 7,23,898 cancer patients in population-based studies (**Supplementary Table 4**), the VTE proportion was 2.9%, whereas among 6,24,242 cancer patients in hospital-based studies (**Supplementary Table 2**), the VTE proportion was 4.1% (see **Supplementary Table 2**). The higher VTE proportion in hospital-based studies suggests an increased risk associated with hospitalization, disease severity, and intensive treatments. This difference was statistically significant as per the Chi-square test, χ²(1, N = 1,348,140) = 1452.22, *p* <.001, indicating a strong association between study setting and VTE occurrence (see **Supplementary Table 3**). Fisher’s exact test and likelihood ratio also supported the significance (p < 0.001).

### VTE proportion across types of cancers: population-based studies

#### Gastrointestinal cancer

**Figure 4:**
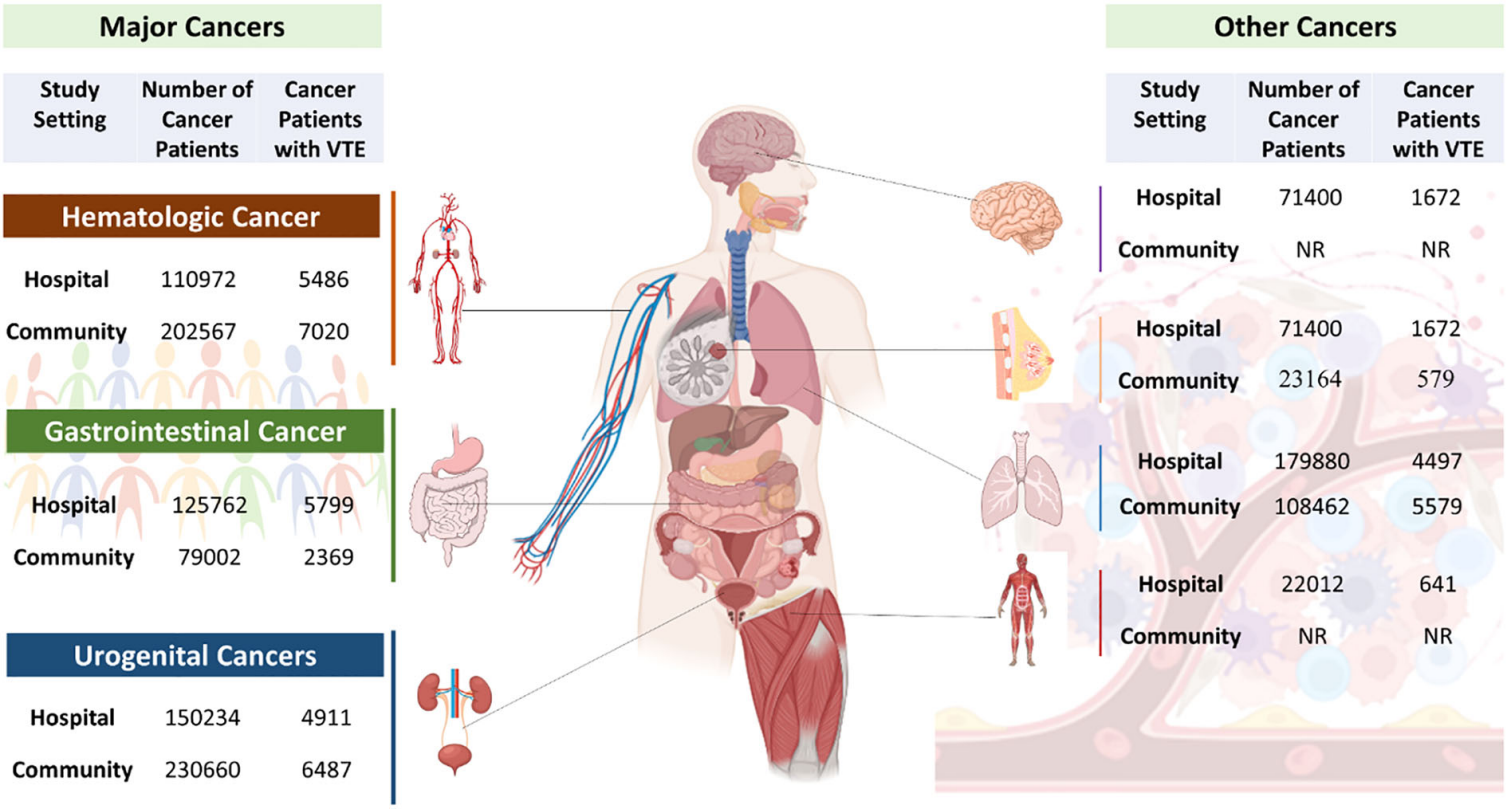
Schematic representation of various cancer types and their association with VTE development. (Created in Biorender.com).

The pooled estimate of VTE proportion among patients with gastrointestinal cancers was 4% (95% CI: 3%–5%), based on a total sample of 202,567 patients. Substantial heterogeneity was observed (I² = 99.3%, τ² = 0.0832, *p* < 0.0001), indicating considerable between-study variability (**Figure 4**) (**Supplementary Figure 4**).

Sensitivity analyses showed that removal of the gastrointestinal cancer reduced the estimated VTE proportion to 3.11% (95% CI: 2.78%–3.48%), with a marked reduction in heterogeneity (I² = 91.9%). In contrast, omission of individual cancer subtypes such as stomach, colon, pancreas, and gall bladder led to marginal increases in the pooled estimates (ranging from 3.53% to 3.73%), while heterogeneity remained consistently high (I² > 99%) (**Supplementary Table 9**).

#### Urogenital cancer

The pooled VTE proportion for urogenital cancers was 3% (95% CI: 2%–3%), with substantial heterogeneity (I² = 92%, τ² = 0.0218, *p* < 0.0001) across 230,660 patients (**Figure 4**) (**Supplementary Figure 5**). Exclusion of individual cancer types yielded modest changes in the pooled estimate: prostate (2.66%), kidney (2.69%), male genital/testes (2.88%), and ovary (2.60%). Particularly, heterogeneity was markedly lower upon removal of male genital/testes cancer (I² = 40.9%), indicating its moderating influence on overall variability (**Supplementary Table 9**).

#### Hematologic cancer

The pooled VTE proportion among hematologic cancer patients was 3% (95% CI: 2.83%–3.34%), with moderate heterogeneity (I² = 71.1%, τ² = 0.0038, *p = 0.0314)* across 79,002 patients (**Figure 4**) (**Supplementary Figure 6**). Sensitivity analysis revealed minimal fluctuation in the pooled estimate upon exclusion of individual subtypes: Non-Hodgkin lymphoma (3.23%), myeloma (2.93%), and leukemia/AML (3.08%). While heterogeneity substantially decreased upon removal of non-Hodgkin lymphoma (I² = 0%) and myeloma (I² = 2.8%), suggesting minimal between-study variability in these subtypes, it remained substantial (I² = 84.7%) when leukemia/AML was excluded, indicating its contribution to variability (**Supplementary Table 9**).

### VTE proportion across types of cancers: hospital-based studies

#### Gastrointestinal cancer

The pooled VTE proportion among gastrointestinal cancer patients was 6% (95% CI: 4%–9%), with substantial heterogeneity (I² = 99.4%, τ² = 0.3605, p < 0.0001) across 110,972 patients (**Supplementary Figure 7**). Sensitivity analysis revealed minimal fluctuation in the pooled estimate upon exclusion of individual subtypes: colorectal cancer (5.89%), stomach (6.24%), pancreas (5.57%), colon (6.47%), and gall bladder (6.74%). Heterogeneity remained high across all subgroups, with I² values ranging from 97.6% to 99.5%, indicating persistent between-study variability irrespective of individual cancer subtype exclusion (**Supplementary Table 10**).

#### Urogenital cancer

Among urogenital cancers, the pooled proportion of VTE was 4% (95% CI: 0.02%–0.06%) based on a combined population of 150,234 patients (**Supplementary Figure 8**). Heterogeneity across studies was extremely high (I² = 99.8%, τ² = 0.2907, *p* < 0.0001), indicating significant variability in the reported proportions. Sensitivity analysis revealed variation across cancer types: the VTE proportion was highest in prostate cancer (4.75%), followed by male genital/testicular cancers (3.94%), and lowest in kidney (3.29%) and ovarian cancers (3.28%). Despite these variations, the overlapping confidence intervals suggest that differences in VTE risk may not be statistically significant. (**Supplementary Table 10**).

#### Hematologic cancer

In hematologic malignancies, the pooled proportion of VTE was estimated at 5% (95% CI: 4%–5%), based on a total sample of 125,762 patients (**Supplementary Figure 9**). The heterogeneity was substantial (I² = 93.4%, τ² = 0.0085, *p* < 0.0001), indicating notable between-study variability. The sensitivity analysis showed closely aligned proportions across Non-Hodgkin Lymphoma (4.58%), Myeloma (4.49%), and Leukemia/AML (4.87%). Despite these similar point estimates, the confidence intervals were narrow and precise, particularly in Leukemia/AML, which likely contributed to the overall robustness of the pooled estimate (**Supplementary Table 10**).

#### Breast cancer

In patients with breast cancer, the pooled proportion of VTE was estimated at 5% (95% CI: 2%–13%), based on a total sample of 71,400 individuals (**Supplementary Figure 10**). Considerable heterogeneity was observed among the studies (I² = 97.0%, τ² = 1.0637, *p* < 0.0001), suggesting substantial between-study variability. Sensitivity analysis revealed variability in individual study estimates, ranging from 3.3% (23) to 6.4% (34), with overlapping yet broad confidence intervals, particularly in studies with smaller sample sizes (24, 32). Heterogeneity remained high in most models (I² > 90%), except for one study (I² = 81.1%) (13), indicating persistent between-study variability (**Supplementary Table 10**).

#### Lung cancer

For lung cancer patients, the pooled estimate of VTE occurrence was 8% (95% CI: 4%–15%), as derived from a combined sample of 108,462 individuals (**Supplementary Figure 11**). There was substantial heterogeneity among the included studies (I² = 95.4%, τ² = 0.5596, *p* < 0.0001), indicating marked variability in effect sizes across studies. Sensitivity analysis showed relatively consistent VTE proportions across individual studies, ranging from 6.5% (23) to 9.2% (22). Heterogeneity remained high in most studies (I² > 95%), with exceptions observed in two studies (I² = 85.2%) (13) and (I² = 82.8%) (26). This suggests persistent between-study variability, potentially attributable to differences in patient characteristics, cancer staging, treatment regimens, or thromboprophylaxis use (**Supplementary Table 10**).

### VTE proportion across stages of cancer

The pooled analysis across cancer stages revealed a non-linear trend in VTE proportions. Specifically, the estimated VTE proportions were 6% for both Stage I and Stage II, increasing to 10% in Stage III and slightly declining to 9% in Stage IV. These findings suggest a higher thrombotic burden in more advanced cancer stages, particularly in Stage III and IV disease. The overall pooled VTE proportion across all stages was 7% (95% CI: 6%–10%), with substantial heterogeneity (I² = 87.6%, τ² = 0.0837, *p* < 0.0001), as illustrated in the forest plot (**Supplementary Figure 12**).

Sensitivity analysis demonstrated that omission of individual stages marginally altered the pooled estimate, ranging from 6.76% to 8.24%, without substantially reducing heterogeneity. For instance, excluding Stage II yielded the highest pooled estimate (8.24%, I² = 78.4%), while excluding Stage III resulted in the lowest (6.76%, I² = 84.7%). These results underscore the robustness of the pooled estimate and highlight Stage III and IV as critical contributors to the elevated VTE risk in cancer progression (**Supplementary Table 11**).

## Discussion

This systematic review and meta-analysis synthesized VTE proportions across a large and diverse global cohort of cancer patients, highlighting significant variation by clinical setting, cancer type, and disease stage. The findings corroborate earlier evidence that tumor characteristics including anatomical sites and stages substantially influence the risk of VTE (35).

The overall VTE proportion was higher in hospital-based studies (4.1%) compared to population-based studies (2.9%). This association was found to be statistically significant as per the chi-square test. likely reflecting the increased thrombotic burden associated with hospitalization, advanced disease, intensive treatment regimens, and procedural interventions. The elevated VTE proportions observed in hospital-based studies are likely multifactorial, stemming from both clinical and demographic factors. Hospitalized cancer patients often present with more advanced disease, undergo aggressive treatment regimens, and are subject to prolonged immobility and invasive procedures—all of which are established risk factors for thrombosis (6).

When stratified by anatomical site, gastrointestinal cancers demonstrated some of the highest VTE proportions, with pooled estimates of 4% and 6% in population- and hospital-based studies, respectively. The sensitivity analyses indicated minimal changes in pooled proportions upon exclusion of individual subtypes, but heterogeneity remained consistently high (I² ≥ 99.3%), suggesting that differences in treatment protocols, staging, and population characteristics may account for inter-study variability. This corroborates earlier findings that gastrointestinal malignancies are highly thrombogenic, owing to tumor biology and treatment-related factors (7). In the case of urogenital cancers, a moderate VTE burden was observed, with pooled proportions of 3% in population-based and 4% in hospital-based studies. Moreover, Thrombocytosis is commonly seen in gastrointestinal, lung, breast, and ovarian cancers, which may lower the threshold for developing VTE (36).

Hematologic malignancies showed pooled VTE proportions of 3% in population-based and 5% in hospital-based cohorts. While overall estimates remained stable during sensitivity analyses, the impact of leukemia/AML on heterogeneity varied by setting, as it reduced variability in population-based studies but contributed substantially to heterogeneity in hospital-based analyses. Among site-specific solid tumors, breast cancer showed a pooled VTE proportion of 5%, with wide confidence intervals and high heterogeneity, particularly in studies with smaller sample sizes. Lung cancer, with a pooled estimate of 8%, emerged as the most thrombotic cancer type analyzed. Individual studies showed consistently high proportions, and heterogeneity persisted even after sensitivity analysis, reinforcing the well-documented association between lung cancer and hypercoagulability (6, 37).

The findings of this study closely align with previous meta-analyses and cohort studies that have reported an increased risk of VTE in gastrointestinal malignancies, which is attributed to both tumor biology and treatment-related factors (6). Moreover, thrombocytosis commonly observed in gastrointestinal, lung, breast, and ovarian cancers has been identified as a key contributor to elevated VTE risk by lowering the thrombotic threshold (13).

The stage-wise analysis demonstrated a clear pattern of increasing VTE risk with advancing cancer stages. Stage I and II showed a pooled proportion of 6%, increasing to 10% in Stage III and slightly decreasing to 9% in Stage IV. These findings reflect the cumulative effect of tumor burden in late-stage disease. Although exclusion of individual stages did not substantially affect the overall pooled estimate (7%), Stage III and IV appeared to contribute more significantly to heterogeneity, highlighting their role as modifiers of thrombotic risk. Numerous studies indicate a clear trend of increasing VTE risk with advancing cancer stages. These results are in agreement with prior research reporting higher VTE recurrence rates among metastatic patients compared to those with localized disease (35). Similarly, another study found that the rate of VTE is likely to be so high in cancer patients due to their late stage of the disease (18). Similarly, in another study, it was reported that patients with high-risk or metastatic cancers had an incidence of 68 per 1,000 person-years, substantially higher than the 13 per 1,000 person-years observed in average-risk patients. Hence, supporting the present results showing elevated VTE proportions in later-stage cancers (38).

The findings of this study align with earlier population-based studies such as those by Walker et al. (2013), which reported elevated VTE incidence in pancreatic, stomach, and lung cancers, consistent with the high pooled proportions observed in our hospital-based cohorts (20). Louzada et al. (2011) further demonstrated increased recurrence rates of VTE in metastatic cancers, which supports our observation of higher VTE proportions in Stage III and IV cancers (35). Unlike prior meta-analyses that focused on specific cancer types or clinical trials, our study offers a broader stratified view, integrating cancer type, stage, and clinical setting. This added granularity supports improved risk stratification and targeted thromboprophylaxis.

The higher VTE burden observed in gastrointestinal, hematologic, and urogenital cancers may be explained by underlying biological mechanisms. For instance, gastrointestinal tumors, especially pancreatic and gastric cancers, are known to express procoagulant factors like tissue factor and mucins (39). Hematologic malignancies may increase thrombotic risk due to immune dysregulation and treatment-related effects (40), while urogenital cancers can promote thrombosis through vascular invasion and paraneoplastic mechanisms (41). In-hospital-based studies likely reflect patients with more advanced disease or intensive treatment exposure, both of which are established VTE risk factors.

The overall quality of the included studies, as assessed using the Joanna Briggs Institute (JBI) tools, ranged from low to moderate risk of bias, with one study rated as high risk (31). Most studies showed strengths in participant selection, exposure measurement, and confounder identification. However, several had methodological limitations such as retrospective design, incomplete reporting of covariates, inconsistent follow-up duration, and variability in cancer staging and VTE diagnostic methods. These issues may have introduced bias and contributed to heterogeneity in effect estimates. The only case-control study (Marks et al., 2014) had moderate risk, performing well in exposure assessment and analysis but lacking clarity in group comparability and confounder control. While a sensitivity analysis excluding the high-risk study did not substantially change the pooled estimates, these limitations underscore the need for cautious interpretation and highlight the importance of standardized, prospective research with comprehensive reporting to strengthen the evidence base on cancer-associated VTE.

### Limitations of the study

While this meta-analysis offers valuable insights, several limitations must be acknowledged. First, notable heterogeneity in study design (hospital- *vs*. population-based) and VTE diagnostic methods may have influenced the pooled estimates. Second, the search strategy included broad terms such as “risk factors” but omitted outcome-specific terms like “incidence” or “proportion,” which may have reduced precision and biased retrieval toward analytical studies. Although only studies reporting VTE incidence or proportion were included, these issues may have affected search comprehensiveness. Additionally, the lack of MeSH in the search may have impacted precision and reproducibility. Citation chaining was also not systematically applied; while relevant reviews were consulted during the scoping phase, their references were not exhaustively screened, potentially limiting the retrieval of additional eligible studies. Substantial heterogeneity remained across pooled estimates, even after stratification by cancer type, stage, and study setting. This likely reflects the influence of unmeasured or inconsistently reported variables such as treatment regimens, thromboprophylaxis practices, and patient-level risk factors. Due to limited reporting, further meta-regression or subgroup analyses were not feasible. Future updates should refine the search strategy by incorporating outcome-specific and MeSH terms, applying citation chaining, and emphasizing the need for standardized reporting in primary studies to support more robust meta-analytic modelling.

Cancer staging harmonization across studies was limited by inconsistent reporting of TNM components. While the TNM system incorporates tumor size (T), nodal involvement (N), and metastasis (M), several included studies reported only partial staging information. In such cases, we conservatively assigned stage groupings using available data, following accepted clinical conventions, as detailed in **Supplementary Table 8**. Although this approach enabled retention of stage as a key stratifying variable, we acknowledge that it introduces potential for staging misclassification. Future studies and meta-analyses would benefit from standardized and complete TNM reporting to support more accurate stage-based comparisons.

Due to the small number of included studies and variability in risk of bias assessment, we were unable to conduct a formal sensitivity analysis excluding high-risk studies or apply quality-weighted models. This limits our ability to quantitatively assess the influence of lower-quality studies such as Grilz et al. (2024) on pooled estimates. Future meta-analyses with larger and more homogeneous datasets may consider integrating quality-based weighting or subgroup exclusion to enhance validity.

Patient demographics, including age, genetic predisposition, and lifestyle factors, were not uniformly accounted for, which may affect generalizability. Additionally, important clinical variables such as cancer-related surgery, chemotherapy, and radiotherapy were inconsistently reported across studies. These treatment-related exposures may have acted as confounding factors, potentially influencing the observed VTE proportions and contributing to between-study variability. Also, as authors, we acknowledge the importance and interpretive utility of the Number Needed to Treat (NNT) in clinical research. The NNT offers a straightforward and intuitive measure of the effectiveness of a healthcare intervention by estimating the number of patients who need to receive a particular treatment for one additional patient to benefit (42). However, NNT is derived from absolute risk reduction (ARR), which presupposes the existence of a control or comparison group, typically in randomized controlled trials (RCTs). Given that the included studies in this review were predominantly observational and not designed as interventional trials, the requisite conditions for calculating NNT were not met. As such, we were unable to compute NNT values within the current meta-analytic framework. Furthermore, the literature search was restricted to a single database (PubMed), which may have limited the comprehensiveness of the evidence base. Future reviews should consider incorporating additional databases to enhance the breadth and representativeness of the included studies. While efforts were made to avoid duplication, two population-based Swedish prostate cancer studies may involve overlapping cohorts. This potential duplication could have inflated the pooled population denominators and VTE event counts, thereby subtly biasing the overall estimates.

## Conclusion

This meta-analysis of observational studies quantified the burden of VTE across cancer types, settings, and stages. Overall, hospital-based studies showed a higher VTE proportion compared to population-based studies. Among cancer categories, gastrointestinal malignancies exhibited the highest pooled VTE proportions, followed by hematologic cancers and urogenital cancers. In hospital-based settings, specific cancers like lung and breast cancers had the highest VTE burden. VTE risk also increased with advancing cancer stage, and was the highest in Stage III and Stage IV. The observed variability across settings and cancer subgroups highlights the need for stage- and type-specific VTE risk assessment models to enhance the precision of strategies to combat VTE. Further prospective studies and multi-center registries are needed to validate findings, develop integrated VTE risk tools (including cancer type, stage, biomarkers, and treatments), and refine prophylactic strategies for high-risk cancer patients.

## Data Availability

The original contributions presented in the study are included in the article/**Supplementary Material**. Further inquiries can be directed to the corresponding author.
