# Supplementary material for: Burden and distribution of venous thromboembolism across cancer types and stages: a meta-analysis of observational studies
Source: Front Oncol. 2025 Sep 30;15:1619554. doi: 10.3389/fonc.2025.1619554 (PMC12518063; doi:10.3389/fonc.2025.1619554)
Supplement: Supplementary file 2 [file DataSheet2.docx]

**Supplementary File 2: Supplementary Tables**

**Supplementary Table 2: Proportion of VTE cases in cancer patients in population-based and hospital-based studies**

| **Study Type** | **Cancer patients (N)** | **Cancer patients with VTE (n)** | **Proportion (%)** |
| --- | --- | --- | --- |
| Population-based | 723898 | 21144.5926 | 2.9 |
| Hospital-based | 624242 | 25763.8874 | 4.1 |

**Supplementary Table 3: Chi-Square of population based and hospital based studies**

|  | **Value** | **df** | **Asymptotic Significance (2-sided)** | **Exact Sig. (2-sided)** | **Exact Sig. (1-sided)** |
| --- | --- | --- | --- | --- | --- |
| **Pearson Chi-Square** | 1452.219^a^ | 1 | .000 |  |  |
| **Continuity Correction^b^** | 1451.860 | 1 | .000 |  |  |
| **Likelihood Ratio** | 1447.265 | 1 | .000 |  |  |
| **Fisher's Exact Test** |  |  |  | .000 | .000 |
| **N of Valid Cases** | 1348140 |  |  |  |  |
|  | | | | | |
|  | | | | | |

**Supplementary Table 4: VTE proportion in cancer patients in population-based studies**

| **Population-Based Studies** | | | |
| --- | --- | --- | --- |
| **Cancer Type** | **Study** | **Number of Cancer Patients** | **Cancer Patients with VTE** |
| **Gastrointestinal** | Grilz et al. | 35490 | 1980.342 |
| **Mesothelium/Soft Tissue** | Grilz et al. | 3897 | 35.073 |
|  | Marks et al. | 4728 | 156.024 |
| **Breast** | Grilz et al. | 23164 | 579.1 |
| **Male Genital, Testes** | Grilz et al. | 21232 | 462.8576 |
| **Lung Cancer** | Marks et al. | 179880 | 4497 |
| **Stomach** | Marks et al. | 22860 | 640.08 |
| **Pancreas** | Marks et al. | 33175 | 1161.125 |
| **Colon** | Marks et al. | 107265 | 3110.685 |
| **Ovary** | Marks et al. | 16112 | 499.472 |
| **Kidney** | Marks et al. | 24611 | 689.108 |
| **Prostate** | Balabanova et al. | 92,105 | 2955 |
|  | Van Hemelrijck et al. | 76600 | 1881 |
| **Gall Bladder** | Marks et al. | 3777 | 128.418 |
| **Non-Hodgkin Lymphoma** | Marks et al. | 55195 | 1600.655 |
| **Myeloma** | Marks et al. | 15318 | 505.494 |
| **Leukemia/AML** | Marks et al. | 8489 | 263.159 |
| **Total** | | **723898** | **21144.5926** |

**Supplementary Table 5: VTE proportion in cancer patients in hospital-based studies**

| **Hospital-Based Studies** | | | |
| --- | --- | --- | --- |
| **Cancer Type** | **Study** | **Number of Cancer Patients** | **Cancer Patients with VTE** |
| Colorectal Cancer | Ikeda et al. | 2477 | 158 |
|  | Vormittag et al. | 111 | 9 |
| Gastrointestinal | Vormittag et al. | 36 | 7 |
| Breast | Khorana et al. | 70917 | 1631.091 |
|  | Al Diab et al. | 165 | 22.79 |
|  | Vormittag et al. | 136 | 2 |
|  | Mandala et al. | 182 | 15.9978 |
| Male Genital, Testes | Khorana et al. | 3687 | 121.671 |
| Lung Cancer | Dimakakos et al. | 217 | 9 |
|  | Khorana et al. | 107587 | 5486.937 |
|  | Al Diab et al. | 45 | 9.89 |
|  | Vormittag et al. | 120 | 6 |
|  | Tagalakis et al. | 493 | 67 |
| Stomach | Khorana et al. | 15934 | 780.766 |
| Pancreas | Khorana et al. | 26118 | 2115.558 |
|  | Vormittag et al. | 47 | 7 |
|  | Mandala et al. | 10 | 0 |
|  | Bloma et al. | 202 | 19 |
| Colon | Khorana et al. | 42258 | 1690.32 |
|  | Al Diab et al. | 73 | 6.02 |
|  | Mandala et al. | 122 | 9.0036 |
| Ovary | Khorana et al. | 23839 | 1334.984 |
|  | Saadeh et al. | 344 | 33 |
| Kidney | Khorana et al. | 29651 | 1660.456 |
|  | Vormittag et al. | 24 | 1 |
| Prostate | Khorana et al. | 92584 | 1759.096 |
|  | Vormittag et al. | 105 | 1 |
| Brain | Khorana et al. | 35297 | 1658.959 |
|  | Vormittag et al. | 103 | 18 |
| Gall Bladder | Khorana et al. | 23584 | 683.936 |
| Sarcoma | Khorana et al. | 21989 | 637.681 |
|  | Al Diab et al. | 23 | 3.225 |
| Non-Hodgkin Lymphoma | Khorana et al. | 56964 | 2734.272 |
| Myeloma | Khorana et al. | 21804 | 1090.2 |
|  | Vormittag et al. | 17 | 1 |
| Leukemia/AML | Khorana et al. | 46977 | 1973.034 |
| **Total** | | **624242** | **25763.8874** |

**Supplementary Table 6: VTE proportion across cancer types in population-based studies**

| **Population Based** | | | | |
| --- | --- | --- | --- | --- |
| **Gastrointestinal** | **Cancer Type** | **Study** | **Number of Cancer Patients** | **Cancer Patients with VTE** |
|  | Gastrointestinal | Grilz et al. | 35490 | 1980 |
|  | Stomach | Marks et al. | 22860 | 640.08 |
|  | Colon | Marks et al. | 107265 | 3110.685 |
|  | Pancreas | Marks et al. | 33175 | 1161.125 |
|  |  |  |  |  |
|  | Gall Bladder | Marks et al. | 3777 | 128.418 |
|  | **Total** | | 202567 | 7020.308 |
| **Urogenital Cancers** | Prostate | Van Hemelrijck et al. and Balabanova et al. | 168705 | 4836 |
|  |  |  |  |  |
|  | Kidney | Marks et al. | 24611 | 689.108 |
|  | Male Genital, Testes | Grilz et al. | 21232 | 462.8576 |
|  | Ovary | Marks et al. | 16112 | 499.472 |
|  | **Total** | | 230660 | 6487.4376 |
| **Hematologic Malignancies** | Non-Hodgkin Lymphoma | Marks et al. | 55195 | 1600.655 |
|  |  |  |  |  |
|  |  |  |  |  |
|  | Myeloma | Marks et al. | 15318 | 505.494 |
|  | Leukemia/AML | Marks et al. | 8489 | 263.159 |
|  | **Total** | | **79002** | **2369.308** |
|  |  |  |  |  |
| **Other Tumors** | Breast | Grilz et al. | 23164 | 579.1 |
|  |  |  |  |  |
|  |  |  |  |  |
|  | Lung Cancer | Marks et al. | 179880 | 4497 |
|  |  |  |  |  |
|  | Mesothelium/Soft Tissue | Grilz et al. | 3897 | 35.073 |
|  |  | Marks et al. | 4728 | 156.024 |
|  |  | |  |  |

**Supplementary Table 7: VTE proportion cases across cancer types in hospital based studies**

| **Hospital Based** | | | | |
| --- | --- | --- | --- | --- |
| **Gastrointestinal** | **Cancer Type** | **Study** | **Number of Cancer Patients** | **Cancer Patients with VTE** |
|  | Colorectal Cancer | Ikeda et al. and Vormittag et al. | 2588 | 167 |
|  |  |  |  |  |
|  | Gastrointestinal | Vormittag et al. | 36 | 7 |
|  | Stomach | Khorana et al. | 15934 | 780.766 |
|  | Pancreas | Khorana et al., Vormittag et al.. Mandala et al., Bloma et al. | 26377 | 2141.558 |
|  |  |  |  |  |
|  |  |  |  |  |
|  |  |  |  |  |
|  | Colon | Khorana et al., Al Diab et al., Mandala et al. | 42453 | 1705.3436 |
|  |  |  |  |  |
|  |  |  |  |  |
|  | Gall Bladder | Khorana et al. | 23584 | 683.936 |
|  |  |  |  |  |
| **Total** | | | **110972** | **5485.6036** |
| **Urogenital Cancers** | Prostate | Khorana et al.and Vormittag et al. | 92689 | 1760.096 |
|  |  |  |  |  |
|  | Kidney | Khorana et al.and Vormittag et al. | 29675 | 1661.456 |
|  |  |  |  |  |
|  | Male Genital, Testes | Khorana et al. | 3687 | 121.671 |
|  | Ovary | Khorana et al. and Saadeh et al | 24183 | 1367.984 |
| **Total** | | | **150,234** | **4911.207** |
| **Hematologic Malignancies** | Non-Hodgkin Lymphoma | Marks et al. | 56964 | 2734.272 |
|  | Myeloma | Khorana et al. and Vormittag et al. | 21821 | 1091.2 |
|  |  |  |  |  |
|  | Leukemia/AML | Khorana et al. | 46977 | 1973.034 |
| **Total** | | | **125762** | **5798.506** |
| **Other Tumors** | Sarcoma | Khorana et al. | 21989 | 637.681 |
|  |  |  |  |  |
|  |  | Al Diab et al. | 23 | 3.225 |
|  | Breast | Khorana et al. | 70917 | 1631.091 |
|  |  | Al Diab et al. | 165 | 22.79 |
|  |  | Vormittag et al. | 136 | 2 |
|  |  | Mandala et al. | 182 | 15.9978 |
|  | Brain | Khorana et al. | 35297 | 1658.959 |
|  |  | Vormittag et al. | 103 | 18 |
|  | Lung Cancer | Dimakakos et al. | 217 | 9 |
|  |  | Khorana et al. | 107587 | 5486.937 |
|  |  | Al Diab et al. | 45 | 9.89 |
|  |  | Vormittag et al. | 120 | 6 |
|  |  | Tagalakis et al. | 493 | 67 |
|  | | |  |  |

**Supplementary Table 8: VTE Proportion in cancer patients across stages**

| **Cancer Stage** | **Original stages** | **Study** | **Type of Cancer** | **Number of Cancer Patients** | **Number of VTE Patients** |
| --- | --- | --- | --- | --- | --- |
| Stage I (Localized: T1-T2, N0, M0) | Stage < IIIb | Tagalakis et al. (2007) | Non-small cell lung cancer | 138 | 12 |
|  | Stage IA1–IB1 | Matsuoa et al. (2020) | Cervical Cancer | 290 | 16 |
|  | Stage I | Saadeh et al. (2013) | Ovarian Cancer | 150 | 6 |
|  | T1 | Mandala et al. (2009) | Breast and Gastrointestinal Cancer | 119 | 7 |
|  | **TOTAL** | | | **697** | **41** |
| Stage II (Early Locally Advanced: T1-T2, N1, M0) | Localized | Vormittag et al. (2009) | Brain 103, Breast 136, Lung 120, Upper gastrointestinal 36,Colorectal 111,  Pancreas 47, Kidney 24, Prostate 105, Multiple myeloma 17, Lymphoma 94 | 301 | 12 |
|  | Limited Stage | Dimakakos et al. (2021) | Small cell lung cancer | 65 | 3 |
|  | Stage II | Saadeh et al. (2013) | Ovarian Cancer | 14 | 0 |
|  | Stage II | Ikeda et al. | Colorectal Cancer | 857 | 49 |
|  | T2 | Mandala et al. (2009) | Breast and Gastrointestinal Cancer | 90 | 10 |
|  | **TOTAL** | | | **1327** | **74** |
| Stage III (Late Locally Advanced: T1-T4, N2-N3, M0) | Stage ≥ IIIb | Tagalakis et al. (2007) | Non-small cell lung cancer | 355 | 55 |
|  | Stage III | Saadeh et al. (2013) | Ovarian Cancer | 149 | 21 |
|  | Stage III | Ikeda et al. | Colorectal Cancer | 1090 | 63 |
|  | Stage IB2–IVA | Matsuoa et al. (2020) | Cervical Cancer | 444 | 63 |
|  | T3 | Mandala et al. (2009) | Breast and Gastrointestinal Cancer | 137 | 8 |
|  | **Total** | | | **2175** | **210** |
| Stage IV (Metastatic: T1-T4, N1-N3, M1) | Extensive Stage | Dimakakos et al. (2021) | Small cell lung cancer | 152 | 6 |
|  | Stage IV | Saadeh et al. (2013) | Ovarian Cancer | 31 | 6 |
|  | Stage IV | Ikeda et al. | Colorectal Cancer | 530 | 46 |
|  | Stage IVB | Matsuoa et al. (2020) | Cervical Cancer | 58 | 17 |
|  | T4 | Mandala et al. (2009) | Breast and Gastrointestinal Cancer | 35 | 3 |
|  | Distant Metastasis | Vormittag et al. (2009) | "Brain 103, Breast 136, Lung 120, Upper gastrointestinal 36,Colorectal 111,  Pancreas 47, Kidney 24, Prostate 105, Multiple myeloma 17, Lymphoma 94 " | 325 | 25 |
|  | **Total** | | | **1131** | **103** |

**Supplementary Table 9. Sensitity Analysis of population studies.**

| **Cancer Category** | **Omitted Subgroup** | **Proportion** | **95% CI** | **p-value** | **τ²** | **I² (%)** |
| --- | --- | --- | --- | --- | --- | --- |
| Gastrointestinal | Gastrointestinal | 0.0311 | [0.0278, 0.0348] | 0.0119 | 0.1089 | 91.9 |
|  | Stomach | 0.0373 | [0.0281, 0.0495] | 0.0876 | 0.296 | 99.4 |
|  | Colon | 0.037 | [0.0276, 0.0496] | 0.0939 | 0.3064 | 99.1 |
|  | Pancreas | 0.0353 | [0.0256, 0.0484] | 0.1111 | 0.3333 | 99.5 |
|  | Gall Bladder | 0.0356 | [0.0260, 0.0485] | 0.1088 | 0.3299 | 99.5 |
|  | Pooled Estimate | 0.0352 | [0.0275, 0.0451] | 0.0833 | 0.2886 | 99.3 |
| Urogenital | Prostate | 0.0266 | [0.0217, 0.0327] | 0.0325 | 0.1802 | 93.9 |
|  | Kidney | 0.0269 | [0.0219, 0.0330] | 0.0337 | 0.1835 | 94.7 |
|  | Male Genital, Testes | 0.0288 | [0.0279, 0.0298] | 0.0002 | 0.0143 | 40.9 |
|  | Ovary | 0.026 | [0.0220, 0.0308] | 0.0225 | 0.1499 | 93.9 |
|  | Pooled Estimate | 0.0272 | [0.0235, 0.0314] | 0.022 | 0.1482 | 92.1 |
| Hematologic | Non-Hodgkin Lymphoma | 0.0323 | [0.0301, 0.0346] | 0 | 0 | 0 |
|  | Myeloma | 0.0293 | [0.0279, 0.0307] | 0.0001 | 0.0081 | 2.8 |
|  | Leukemia/AML | 0.0308 | [0.0271, 0.0349] | 0.0075 | 0.0864 | 84.7 |
|  | Pooled Estimate | 0.0307 | [0.0283, 0.0334] | 0.0038 | 0.0613 | 70.8 |

**Supplementary Table 10. Sensitity Analysis of hospital studies.**

| **Cancer Category** | **Omitted Subgroup** | **Proportion** | **95% CI** | **p-value** | **τ²** | **I² (%)** |
| --- | --- | --- | --- | --- | --- | --- |
| Gastrointestinal | Colorectal Cancer | 0.0589 | [0.0322, 0.1052] | 0.4899 | 0.6999 | 99.5 |
|  | Gastrointestinal | 0.0497 | [0.0348, 0.0705] | 0.1782 | 0.4221 | 99.5 |
|  | Stomach | 0.0624 | [0.0342, 0.1110] | 0.486 | 0.6972 | 99.5 |
|  | Pancreas | 0.0557 | [0.0314, 0.0967] | 0.4342 | 0.6589 | 97.6 |
|  | Colon | 0.0647 | [0.0366, 0.1119] | 0.4392 | 0.6627 | 99.4 |
|  | Gall Bladder | 0.0674 | [0.0429, 0.1044] | 0.2714 | 0.521 | 99.3 |
|  | Pooled Estimate | 0.059 | [0.0368, 0.0933] | 0.3612 | 0.601 | 99.4 |
| Urogenital | Prostate | 0.0475 | [0.0337, 0.0666] | 0.0971 | 0.3117 | 94.4 |
|  | Kidney | 0.0329 | [0.0175, 0.0608] | 0.3211 | 0.5666 | 99.8 |
|  | Male Genital, Testes | 0.0394 | [0.0193, 0.0787] | 0.4211 | 0.6489 | 99.9 |
|  | Ovary | 0.0328 | [0.0176, 0.0603] | 0.3153 | 0.5615 | 99.8 |
|  | Pooled Estimate | 0.0376 | [0.0225, 0.0624] | 0.291 | 0.5394 | 99.8 |
| Hematologic | Non-Hodgkin Lymphoma | 0.0458 | [0.0386, 0.0543] | 0.0159 | 0.1263 | 95.5 |
|  | Myeloma | 0.0449 | [0.0394, 0.0512] | 0.0093 | 0.0965 | 95.3 |
|  | Leukemia/AML | 0.0487 | [0.0469, 0.0506] | 0.0002 | 0.0158 | 27 |
|  | Pooled Estimate | 0.0465 | [0.0419, 0.0515] | 0.0084 | 0.0919 | 93.4 |
| **Cancer Type** | **Studies** | **Proportion** | **95% CI** | **p-value** | **τ²** | **I² (%)** |
| Breast | Khorana et al. | 0.0644 | [0.0198, 0.1901] | 0.9975 | 0.9988 | 81.1 |
|  | Al Diab et al. | 0.0333 | [0.0120, 0.0890] | 0.7008 | 0.8371 | 92 |
|  | Vormittag et al. | 0.0631 | [0.0216, 0.1706] | 0.9329 | 0.9659 | 97.8 |
|  | Mandala et al. | 0.0391 | [0.0098, 0.1427] | 1.3931 | 1.1803 | 97 |
|  | Pooled Estimate | 0.0484 | [0.0176, 0.1259] | 1.0009 | 1.0004 | 96.7 |
| Lung | Dimakakos et al. | 0.0923 | [0.0460, 0.1767] | 0.5074 | 0.7123 | 96.4 |
|  | Khorana et al. | 0.0906 | [0.0423, 0.1835] | 0.5823 | 0.7631 | 85.2 |
|  | Al Diab et al. | 0.0647 | [0.0360, 0.1138] | 0.3325 | 0.5766 | 95.5 |
|  | Vormittag et al. | 0.0878 | [0.0416, 0.1757] | 0.5964 | 0.7723 | 96.4 |
|  | Tagalakis et al. | 0.0679 | [0.0328, 0.1352] | 0.5084 | 0.7131 | 82.8 |
|  | Pooled Estimate | 0.0797 | [0.0426, 0.1441] | 0.4957 | 0.7041 | 95.2 |

**Supplementary Table 11. Sensitivity Analysis of stages**

| **Omitted Stage** | **Proportion** | **95% Confidence Interval** | **p-value** | **τ²** | **I² (%)** |
| --- | --- | --- | --- | --- | --- |
| Stage 1 | 0.0797 | [0.0568, 0.1105] | 0.0923 | 0.3038 | 89.2 |
| Stage 2 | 0.0824 | [0.0620, 0.1087] | 0.0602 | 0.2454 | 78.4 |
| Stage 3 | 0.0676 | [0.0490, 0.0926] | 0.0746 | 0.2731 | 84.7 |
| Stage 4 | 0.0693 | [0.0481, 0.0988] | 0.1025 | 0.3201 | 91.2 |
| Pooled Estimate | 0.0746 | [0.0560, 0.0986] | 0.0837 | 0.2894 | 87.6 |
