# Supplementary material for: Burden and distribution of venous thromboembolism across cancer types and stages: a meta-analysis of observational studies
Source: Front Oncol. 2025 Sep 30;15:1619554. doi: 10.3389/fonc.2025.1619554 (PMC12518063; doi:10.3389/fonc.2025.1619554)
Supplement: Supplementary file 3 [file DataSheet3.docx]

**Supplementary File 3: Supplementary Figures**

**Supplementary Fig 4. Pooled proportion of venous thromboembolism for gastrointestinal cancer in population-based studies.**


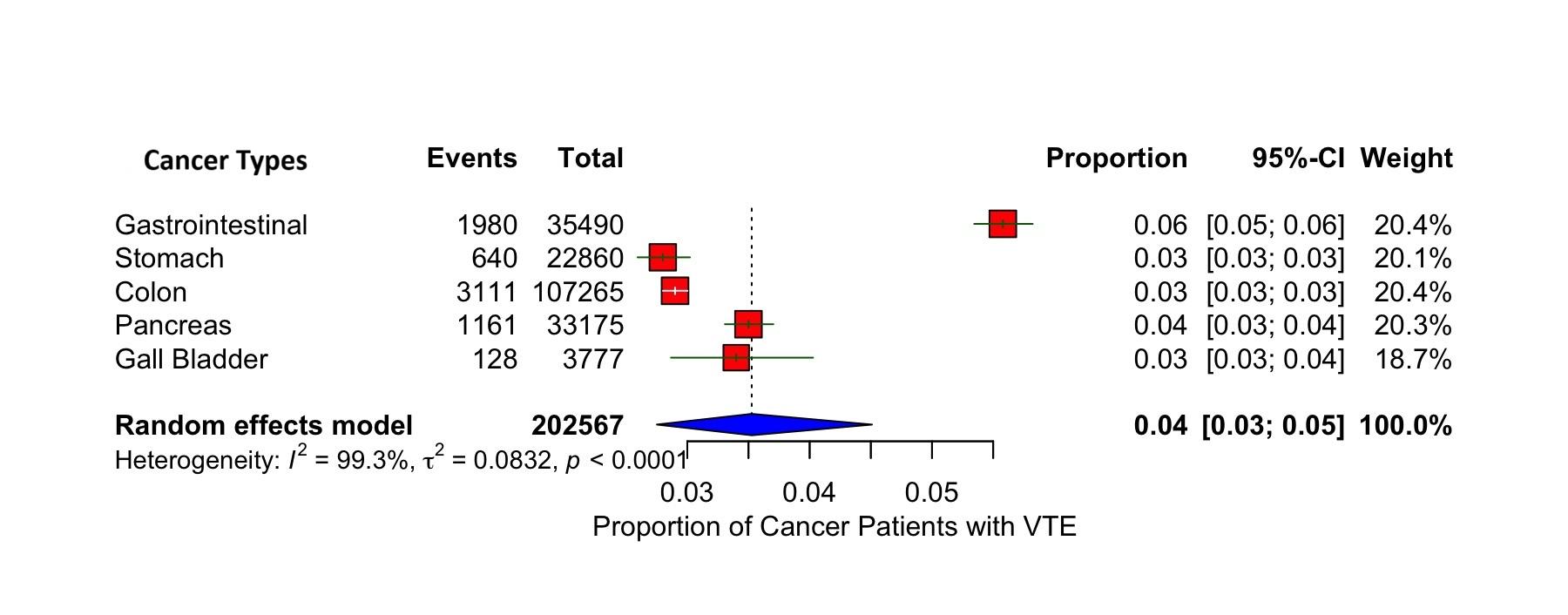


**Supplementary Fig 5. Pooled proportion of venous thromboembolism for urogenital cancer in population-based studies.**


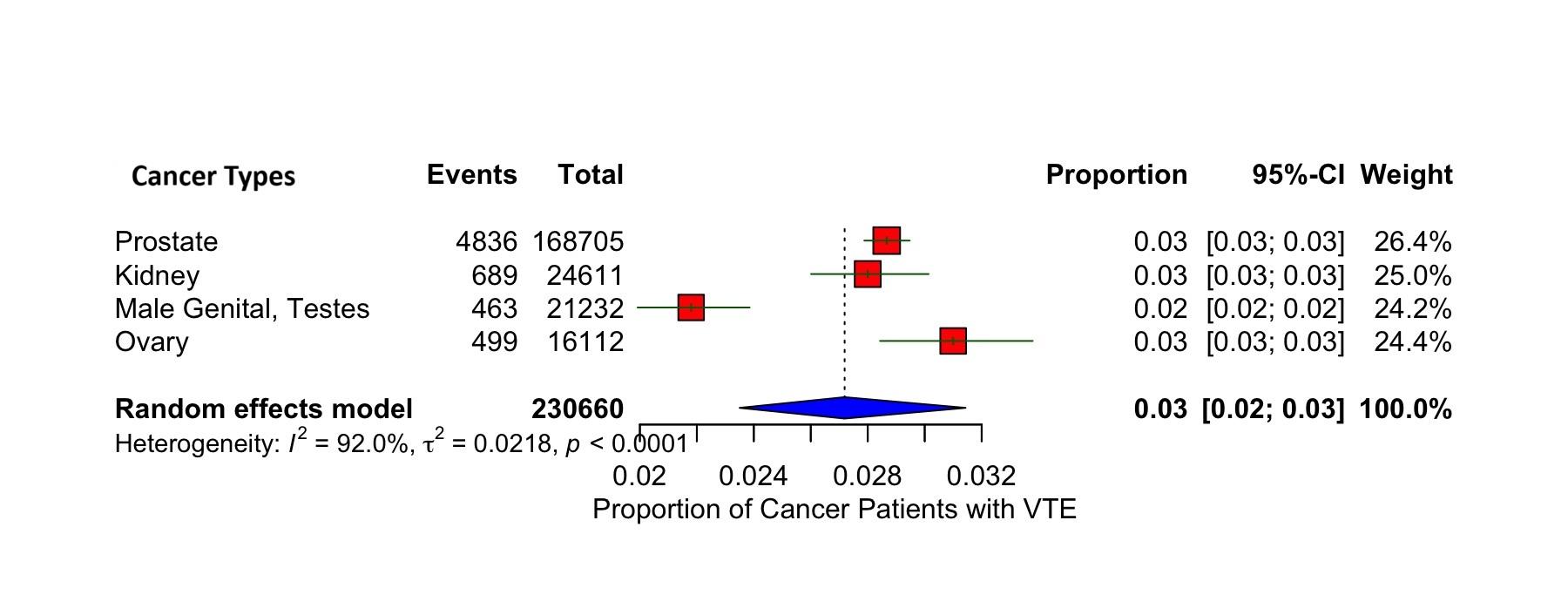


**Supplementary Fig 6. Pooled proportion of venous thromboembolism for hematologic cancer in population-based studies.**

***
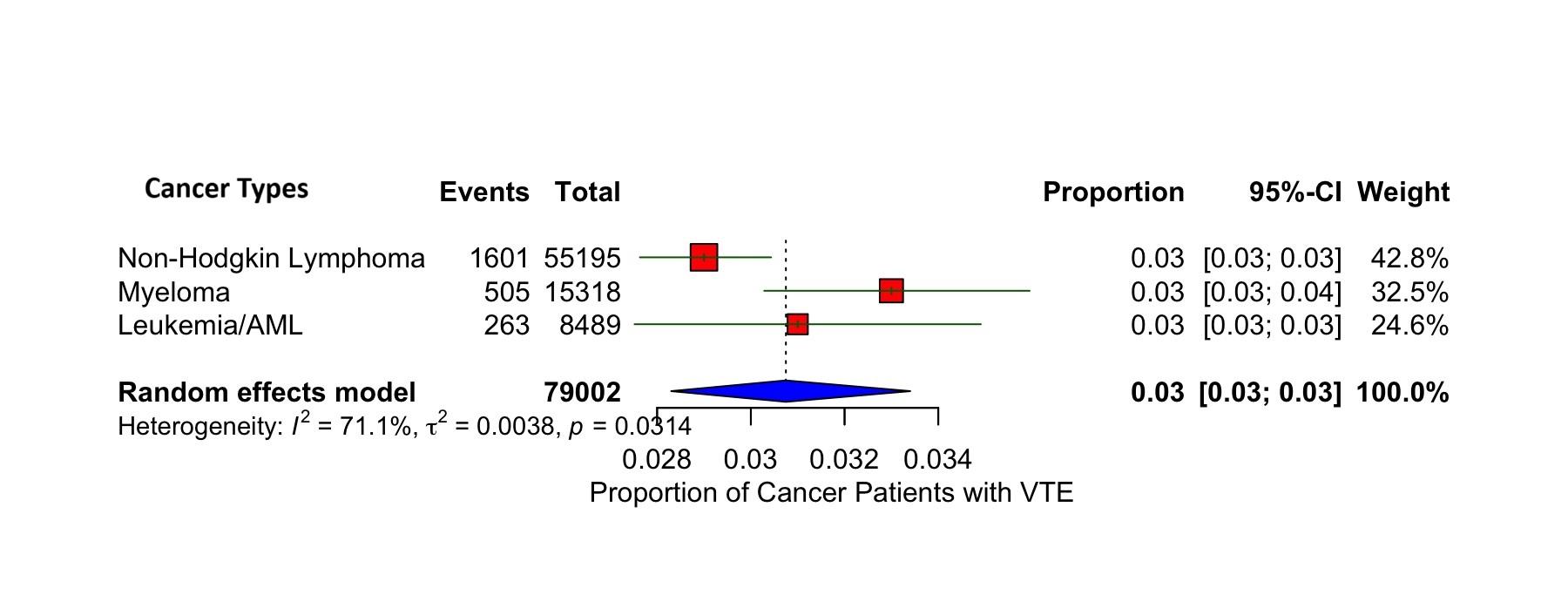
***

**Supplementary Fig 7. Pooled proportion of venous thromboembolism for gastrointestinal cancer in hospital studies.**

***
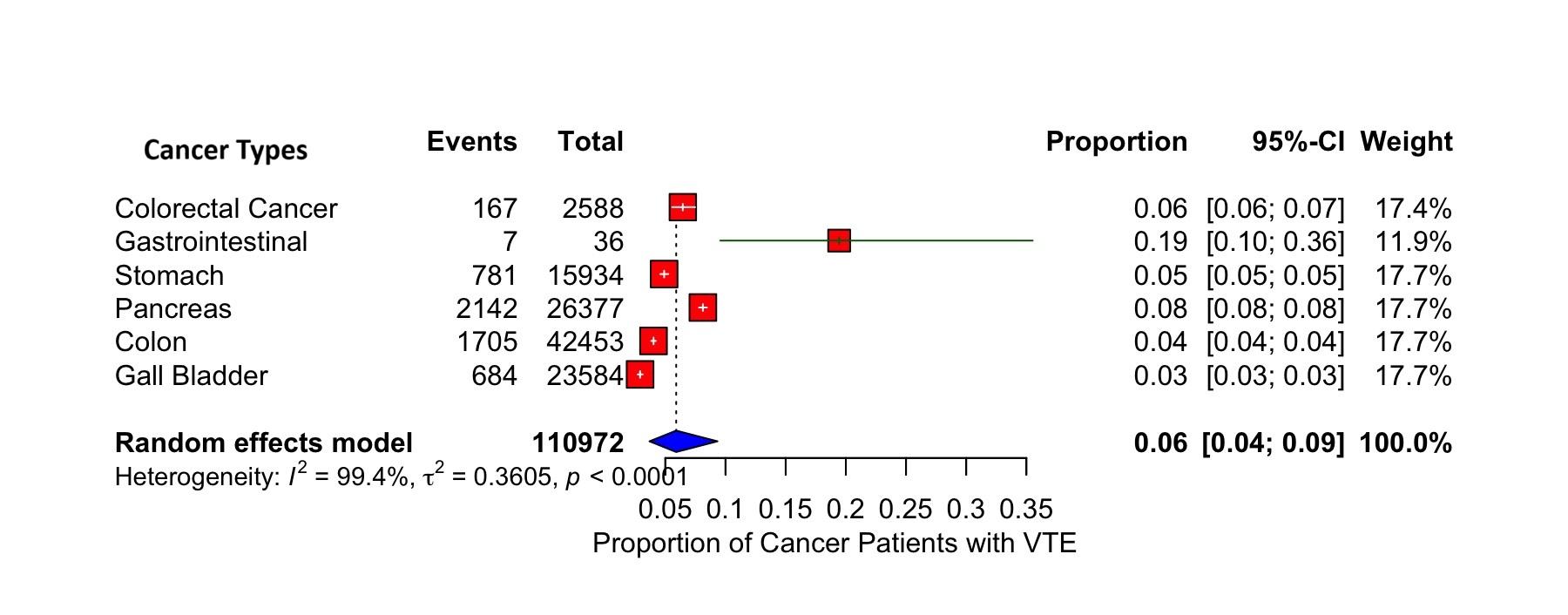
***

**Supplementary Fig 8. Pooled proportion of venous thromboembolism for urogenital cancer in hospital studies.**

***
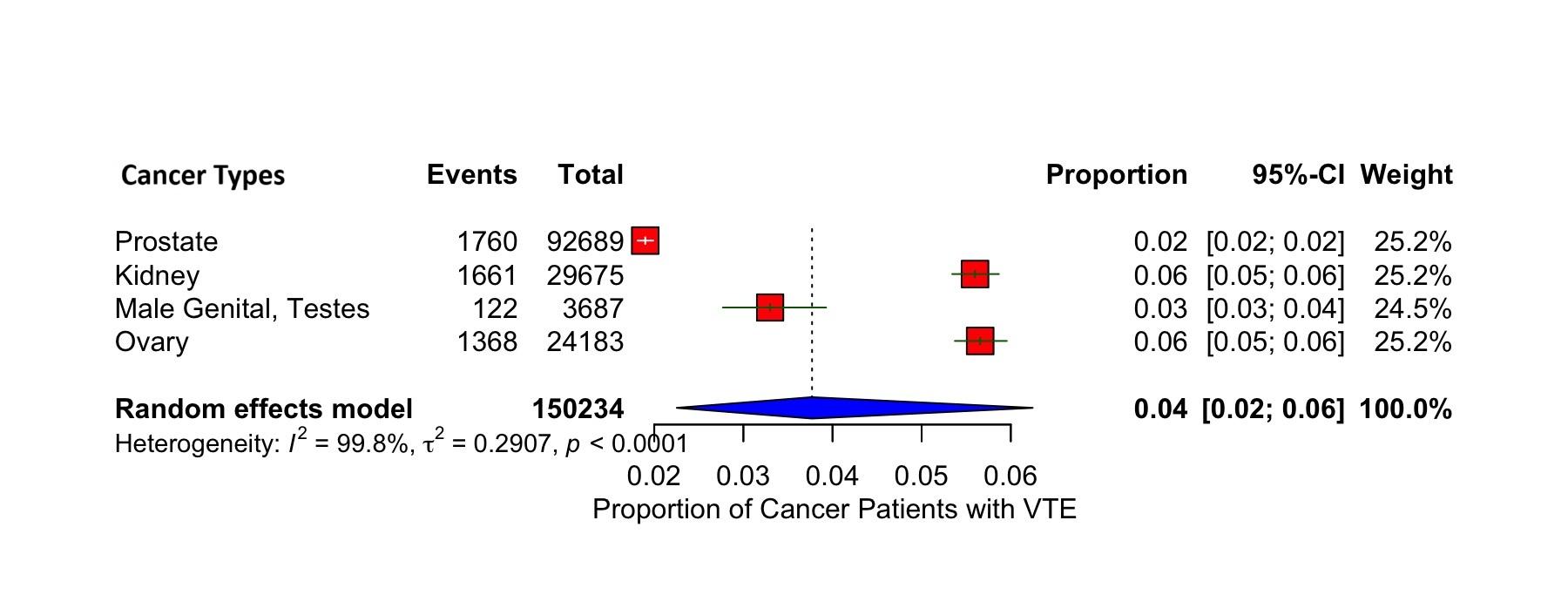
***

**Supplementary Fig 9: Pooled proportion of venous thromboembolism for hematologic cancer in hospital studies.**

**
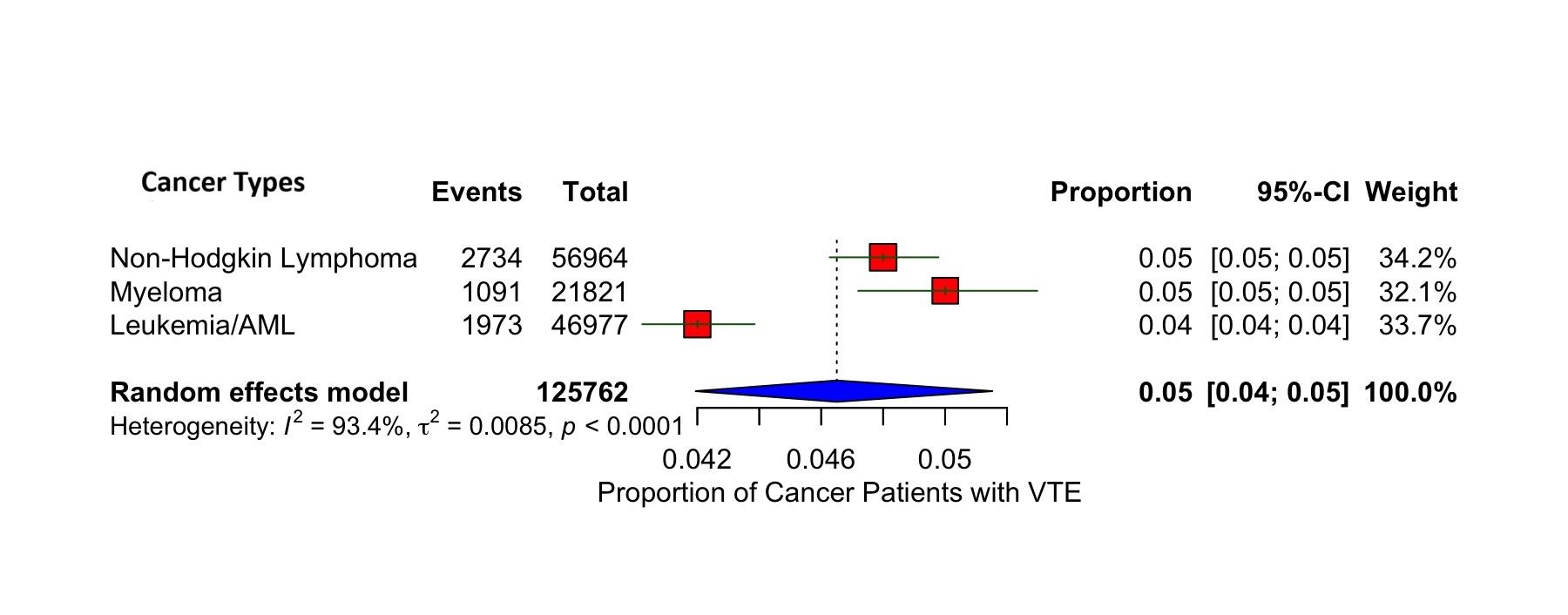
**

**Supplementary Fig 10. Pooled proportion of venous thromboembolism for breast cancer in hospital studies.**

***
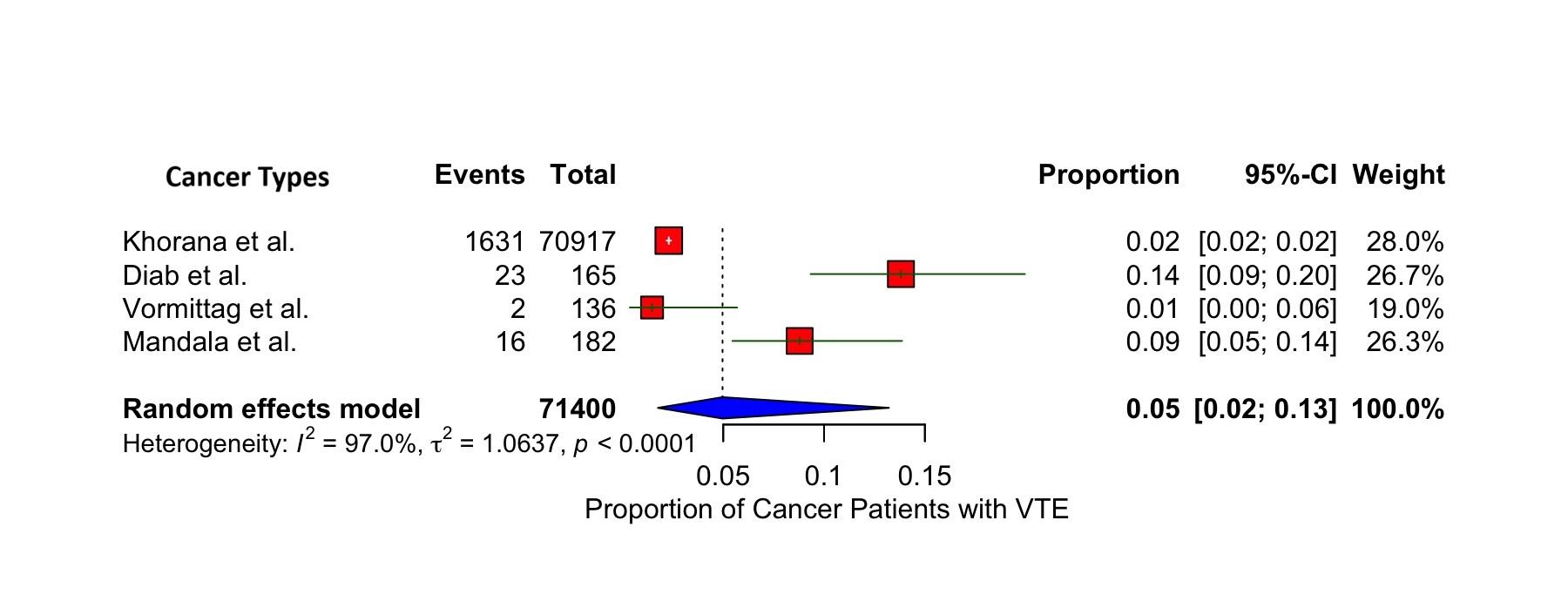
***

**Supplementary Fig 11. Pooled proportion of venous thromboembolism for lung cancer in hospital studies.**

***
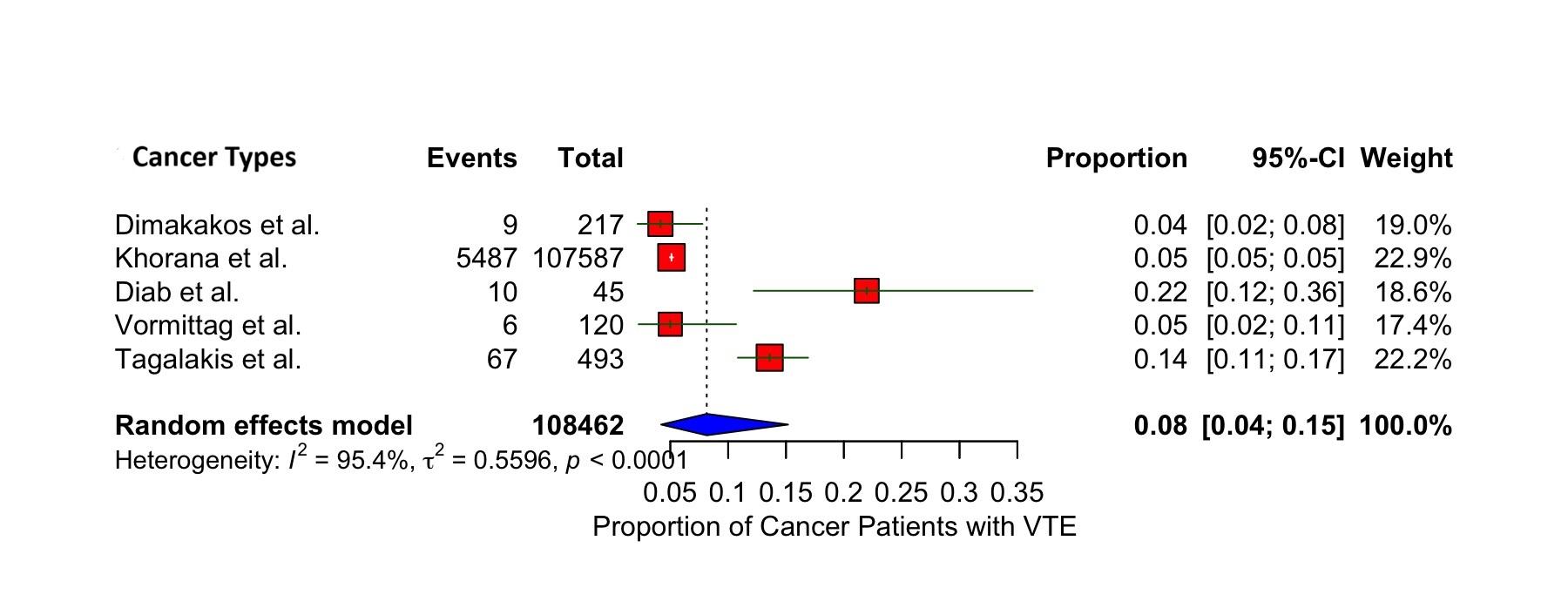
***

**Supplementary Fig 12. Cancer stage-wise forest plot.**

**
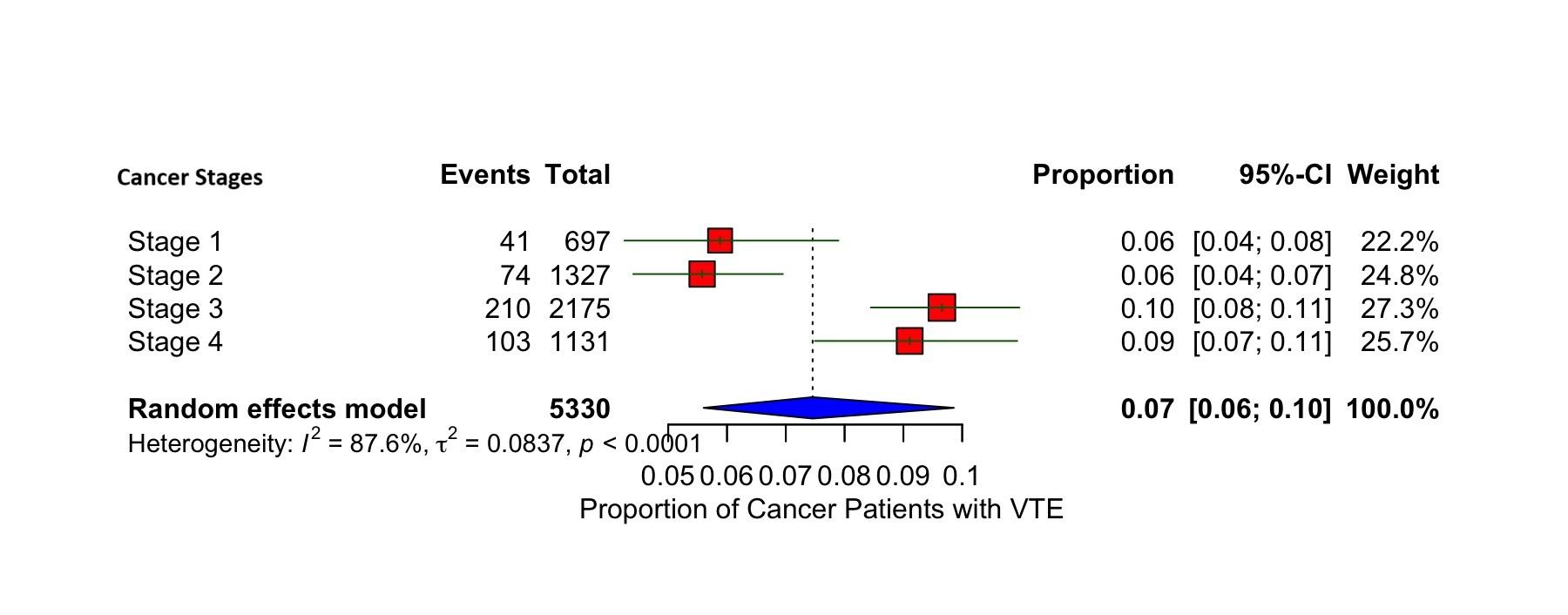
**
